# Measurement of Fruit and Vegetable Intake Incorporating a Diversity, Equity, and Inclusion Lens. Comment on Di Noia, J.; Gellermann, W. Use of the Spectroscopy-Based Veggie Meter^®^ to Objectively Assess Fruit and Vegetable Intake in Low-Income Adults. *Nutrients* 2021, *13*, 2270

**DOI:** 10.3390/nu14040809

**Published:** 2022-02-15

**Authors:** Carmen Byker Shanks, Betty Izumi, Courtney A. Parks, Amy L. Yaroch

**Affiliations:** 1 Gretchen Swanson Center for Nutrition, Omaha, NE 58154, USA; cparks@centerfornutrition.org (C.A.P.); ayaroch@centerfornutrition.org (A.L.Y.); 2 Department of Health and Human Development, Montana State University, Bozeman, MT 59717, USA; 3 OHSU-PSU School of Public Health, Oregon Health and Science University-Portland State University, Portland, OR 97201, USA; izumibet@pdx.edu

Disparities in fruit and vegetable intake (FVI) and diet-related diseases exist among low-income and racial/ethnic minority populations [1,2,3,4]. Intervention approaches to eliminate FVI disparities frequently utilize dietary assessment to measure impact. Studies measure FVI in varying ways, but do not fully account for diversity, equity, and inclusion (DEI).

Di Noia and Gellerman upheld the Veggie Meter using resonance Raman spectrometry as a non-invasive, objective biomarker approach to measure FVI for assessing dermal carotenoids among low-income populations [5]. However, a meta-analysis reported that the validity of resonance Raman spectrometry has mainly been established among White populations, and research among non-White populations is warranted [6].

Dietary recalls assess FVI by capturing a variety of self-reported foods [7]. Recall data are entered into food analysis software, and these data are limited by the foods available in the database. Researchers find substitute foods when the participant’s response is not in the database. For example, the fruit atemoya is consumed across cultures, but not found in most databases.

Screeners are frequently employed in intervention studies that target FVI, since they are brief, inexpensive, and assess defined variables of interest [8]. Screeners are limited by items assessed within a food group (e.g., tomato sauce represents the red/orange vegetable subgroup) or example foods (e.g., delimiting labeling such as “Mexican-type salsa with tomato”).

FVI is an indicator of a behavior and, if interpreted out of context, can be used to place blame on the individual for poor dietary choices. Pairing FVI with other known data sources (e.g., food access) can shift the conversation to focus on systematic and external barriers that influence dietary patterns among populations.

Sociodemographic categories also may not reflect actual identities. In the U.S., FVI measures are typically validated in English, and should be expanded to other languages to ensure the relevance of translations. Questions based upon sex/gender customarily ask about the female and male genders, and do not extend to non-binary, third gender, and/or self-described options. Racial/ethnic groups are typically limited to five categories specified in the 1997 Office of Management and Budget (OMB) standards on race and ethnicity, and do not address the heterogeneity among groups in responsive and inclusive ways [9].

FVI and related data are potentially powerful tools to illuminate systemic inequities, but they are only as useful as their applicability across populations. Measures integrating diversity (the ways in which individuals differ) could ensure the inclusion of the variety/types of FVs consumed to reflect the intake of diverse populations. Measures integrating equity (equal access to opportunities) could measure factors that drive FVI. Measures integrating inclusion (including all individuals) could address sociodemographic variables and languages with which people identify. The ability to understand the effectiveness of interventions in eliminating disparities in FVI is dependent upon measures adequately representing dietary patterns and influencing factors, but this is not feasible without incorporating DEI.
